# Lizards and rabbits may increase Chagas infection risk in the Mediterranean-type ecosystem of South America

**DOI:** 10.1038/s41598-020-59054-8

**Published:** 2020-02-05

**Authors:** Esteban San Juan, Raúl Araya-Donoso, Alejandra Sandoval-Rodríguez, Andrea Yáñez-Meza, Nicol Quiroga, Carezza Botto-Mahan

**Affiliations:** 1 Departamento de Ciencias Ecológicas, Facultad de Ciencias, Universidad de Chile, P.O. Box 653 Santiago, Chile; 2 Departamento de Medicina Preventiva, Facultad de Ciencias Veterinarias y Pecuarias, Universidad de Chile, Santiago, Chile

**Keywords:** Community ecology, Ecological epidemiology

## Abstract

Studies of host-parasite relationships largely benefit from adopting a multifactorial approach, including the complexity of multi-host systems and habitat features in their analyses. Some host species concentrate most infection and contribute disproportionately to parasite and vector population maintenance, and habitat feature variation creates important heterogeneity in host composition, influencing infection risk and the fate of disease dynamics. Here, we examine how the availability of specific groups of hosts and habitat features relate to vector abundance and infection risk in 18 vector populations along the Mediterranean-type ecosystem of South America, where the kissing bug *Mepraia spinolai* is the main wild vector of the parasite *Trypanosoma cruzi*, the etiological agent of Chagas disease. For each population, data on vectors, vertebrate host availability, vegetation, precipitation, and temperature were collected and analyzed. Vector abundance was positively related to temperature, total vegetation, and European rabbit availability. Infection risk was positively related to temperature, bromeliad cover, and reptile availability; and negatively to the total domestic mammal availability. The invasive rabbit is suggested as a key species involved in the vector population maintenance. Interestingly, lizard species –a group completely neglected as a potential reservoir–, temperature, and bromeliads were relevant factors accounting for infection risk variation across populations.

## Introduction

Studies of host-parasite relationships largely benefit from adopting a multifactorial approach, including the complexity of multi-host systems and habitat features in their analyses^1,2^. For instance, because of their different susceptibility to acquire and propagate infection, not all host species have similar roles in infectious disease dynamics. Specifically, host reservoir species are expected to play a critical role as they often concentrate most of infection and have a disproportionate impact on parasite and vector populations^3,4^. While the impact of reservoir species on disease dynamics is a focus of important research at present^5^, most studies conducted to date have been carried out at the local spatial scale of analysis^6,7^ and few examine the extent to which the geographical turnover in host species composition influence infection risk^8^. This lack of emphasis is unfortunate as variation in species assemblages is the norm rather than the exception in ecological studies and variation in climatic factors is a major determinant of infectious disease dynamics^9^. In this way, habitat feature variation across localities is expected to create important heterogeneity in the type of host species, influencing the prevalence, infection risk and ultimately the fate of disease dynamics.

In this study, we assess how specific host assemblages and habitat features relate to vector abundance and infection risk (measured as infected vector abundance) in a range of abiotic conditions across populations. We focus on a system composed of the flagellated protozoan parasite *Trypanosoma cruzi* (the causative agent of Chagas disease), its hematophagous triatomine vector (kissing bugs), and vertebrate assemblages that include reptiles, birds, and native and non-native mammals from the Mediterranean-type ecosystem of South America (Chile). The climate of this ecosystem is of a semiarid Mediterranean type with most rainfall occurring in the winter season, with a mean annual rainfall between 25 and 700 mm depending on the latitude^10^. Vegetation is dominated by succulents, shrubs, and sclerophyllous species. Only mammal species have been identified as *T. cruzi* reservoirs and, in principle, they could contribute differently to infection maintenance since native mammals may be competent reservoirs as the result of their evolutionary history with *T. cruzi* and kissing bugs, while domestic mammals have a shorter history of interaction with kissing bug species and *T. cruzi*^5,8^.

**Figure 1:**
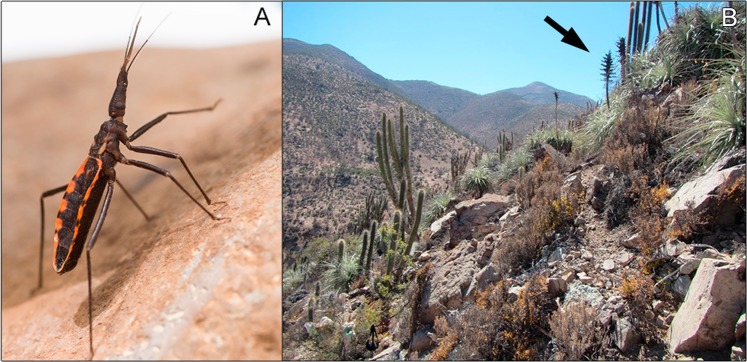
(**A**) Micropterous adult male of *Mepraia spinolai* showing its extended proboscis. (**B**) Arborescent scrub habitat with predominance of bromeliads (*Puya* sp. highlighted by a black arrow).

In this ecosystem, the most important kissing bug species is the wild triatomine *Mepraia spinolai*^11^ (Fig. 1A). This diurnal kissing bug species occurs from 26°S to 33°S^12^ and shows a conspicuous spatial aggregation, often using rocky outcrops and bromeliads as refuge (Fig. 1B), where it exhibits a sit-and-wait strategy to parasitize vertebrate hosts^7,13–16^. *Mepraia spinolai* feeds on the blood of vertebrates such as mammals, birds and reptiles^17,18^, albeit its preferred hosts consist on several small native mammals, reaching *T. cruzi* infection prevalence up to 70% in some rodent species^16,19^. Secondarily, the European rabbit *Oryctolagus cuniculus*, an invasive mammal present in this ecosystem since 1884, experiences *T. cruzi* infection prevalence up to 38%^20^. Overall, only 22 native mammal species overlap with *M. spinolai* geographic distribution; therefore, it is epidemiologically relevant to examine the role of non-native mammal species (free-ranging and domestic) in this mammal-depauperate ecosystem.

The first goal of our study was to identify the vertebrate assemblages that influence vector abundance and the maintenance of the protozoan *T. cruzi* at regional scale. Because habitat features such as temperature, precipitation and vegetation cover, often affect host and insect vector demographic parameters such as survival and reproductive rates^21–23^, the infection risk found in natural populations may be also determined by variation in these factors^24,25^. Our second goal was to examine the importance of habitat features on vector abundance and infection risk at the regional scale. More specifically, in this study we address the following questions: (1) Do reptiles, birds, and mammals influence vector abundance and infection risk at the regional scale? (2) To what extent are these effects - if any - modulated by the habitat features? (3) What is the importance of non-native mammal species on vector abundance and infection risk across localities?

## Materials and Methods

### Field procedures

**Figure 2:**
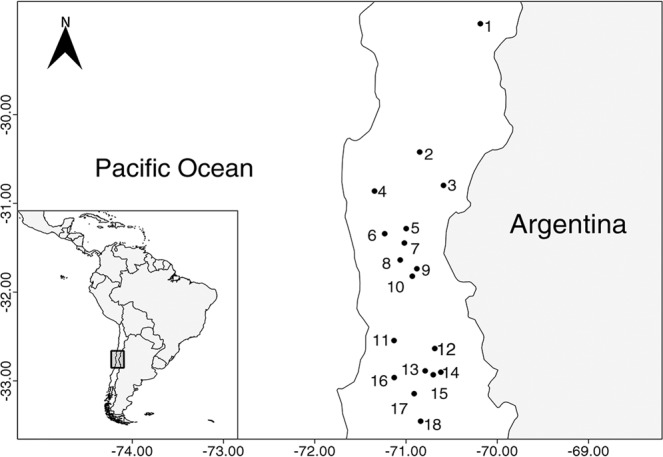
Map of north-central Chile with the geographic distribution of the 18 sampled populations of *Mepraia spinolai*.

The study was carried out in 18 study sites encompassing a range of 500 km, between 28°58′ and 33°27′S (see Table 1; Fig. 2). During the austral summer, specimens of all stages of *M. spinolai* were collected at each study site (7 sites in 2015; 11 sites in 2016) by 3–4 trained researchers between 1100 and 1600 h the time of day with maximum *M. spinolai* activity^14^. Each site was geo-referenced in UTM coordinates (precision: ± 3 m) using a handheld GPS device. The collected kissing bugs were classified according their stage of development and individually stored to avoid potential cross-contamination with *T. cruzi* - infected feces. In the laboratory, kissing bugs were euthanized and subjected to abdominal extrusion to obtain a sample of its intestine and feces for subsequent DNA analyses. Each sample was diluted with 200 μL of double-distilled water. Whole genomic DNA was isolated from fecal samples (MOBIO, UltraClean Tissue & Cell DNA DNA Isolation Kit) and stored at −20 °C until molecular analyses. The Ethical Committee of the Faculty of Science of the University of Chile, and the National Forest Corporation (CONAF) reviewed and approved the animal-handling protocol for this study.

**Table 1:** Descriptive information of the 18 populations of *Mepraia spinolai* under study.

Site	Location	Latitude	Longitude	MS/h	IMS/h	TAP (mm)	TWT (°C)	VC	BC	NMA	DMA	OCA	BA	RA
1	Los Tambos	28°58.6S	70°11.2W	7.05	3.04	40.4	16.6	0.21	0	19.3	0	0	2.3	0.7
2	Pichasca	30°25.3S	70°51.0W	4.2	4	53	18	0.3	0	0.7	0	0	0.7	1.3
3	El Maitén	30°47.8S	70°35.4W	8.22	0.23	95.4	18.2	0.4	0	4.3	1.7	0	4.7	2.3
4	La Rinconada	30°51.7S	71°20.7W	3.87	0.06	127.5	18.4	0.21	0	4.7	0	0	0	0
5	Valle Hermoso	31°17.0S	70°59.9W	9.59	0.12	82.1	17.3	0.37	0	4	0	0	1.3	0
6	Los Pozos	31°20.5S	71°14.0W	19.78	17.94	202.7	18.3	0.56	0.43	3.3	3.3	1	2	1
7	Farellón Sánchez	31°26.8S	71°1.1W	3.31	1.4	272.2	18.4	0.19	0.01	27.3	0	0	0.7	1
8	Caña de Michío	31°38.2S	71°3.8W	13.25	12.52	102.1	18.2	0.42	0.13	60.3	0	0	4.7	3.3
9	San Agustín	31°44.2S	70°52.9W	20.5	19.4	263	18.8	0.79	0.06	0.7	0.3	0	1.7	6
10	La Higuerilla	31°49.2S	70°55.9W	20.9	18.2	270	20	0.72	0.07	10	0	0	1.5	1.5
11	La Patagua	32°32.7S	71°7.9W	13.3	12.1	344	19.4	0.51	0.01	12.7	0.3	1.7	22	1
12	Sahondé	32°38.1S	70°41.1W	11	3.53	204	18.9	0.51	0	19	3.7	3.3	36	0.7
13	Las Blancas	32°53.2S	70°47.4W	9.06	6.09	255	18.1	0.55	0.06	4.7	0	0.3	11.7	0.7
14	Pedrero	32°54.1S	70°37.1W	22	20.77	200.9	19.6	0.64	0	2.3	2	1.3	8	4.7
15	Chacabuco	32°55.8S	70°42.1W	16.14	15.98	260.7	17.8	0.55	0.27	196.7	0	8.3	10.3	11.3
16	La Campana	32°57.7S	71°7.8W	8.21	5.62	422	15.9	0.75	0.39	28	0.3	0	6	3
17	Til Til	33°8.6S	70°54.7W	23.05	7.62	81.8	19.9	0.79	0.16	7.7	6.7	1.3	19.3	5
18	Ciudad de los Valles	33°27.2S	70°50.4W	15.21	7.24	161.6	19.9	0.39	0	0.7	0.7	4.7	20	0

MS/h: number of *M. spinolai* per hour; IMS/h: number of infected *M. spinolai* per hour; TAP: total annual precipitation; TWT: mean temperature of the warmest trimester; VC: vegetation cover; BC: bromeliad cover; NMA: mean number of native mammal recordings by month, DMA: mean number of domestic mammal recordings by month; OCA: mean number of *Oryctolagus cuniculus* recordings by month; BA: mean number of bird recordings by month; RA: mean number of reptile recordings by month.

For each vector population, mammal, bird and reptile species composition and availability were sampled via camera trapping (Bushnell Nature View HD Max) during peak kissing bug abundance from December to March (i.e., austral summer). Camera traps were placed to include the action area of the vector population. All photographic records were checked and classified using specific field guides for each vertebrate taxonomic group (mammals, birds, and reptiles). On this basis, the availability of each vertebrate group at each vector population was calculated as the mean number of records per month. In addition, we subdivided the mammals based on native (including all rodent, carnivore, and didelphimorph species), non-native feral (including the European rabbit), and non-native domestic (including all livestock, dogs and cats). Because we were mostly interested in reflecting blood meal availability to *M. spinolai*, instead of enumerating the individuals within each vertebrate species, repeated records of the same individual of vertebrate species were meaningful measures from the kissing bug’s perspective. To avoid the overestimation of availability, we considered photo records of the same species separated by one-hour interval.

In the surrounding of each vector population, the vegetation was sampled by means of three transects of 50 m each. Vegetation cover was calculated as the proportion of total vegetation on the three transects altogether, including herbs, succulents, shrubs and trees. Additionally, the specific cover of bromeliads (*Puya* sp.; Fig. 1B) was calculated because of the reported role of these plant species as kissing bug shelter^15^.

Regarding climatic variables, the mean temperature of the warmest trimester was obtained using the bioclimatic models developed by Pliscoff^10^ in the QGIS 2.18.14^26^ software using the “Point sampling tool” complement. Likewise, the total annual precipitation of the previous year of kissing bug collection - recorded by the nearest climatic station to each population (8.4 km distant on the average) - was downloaded from the Center of Climate and Resilience Research CR2 database (http://www.cr2.cl/recursos-y-publicaciones/bases-de-datos/). In this way, under any association, data on kissing bug abundance and infection would respond to rainfall at the appropriate temporal scale.

### Molecular procedure for *Trypanosoma cruzi* detection

The amplification reaction for fecal samples was performed in triplicate with oligonucleotides 121 (5′-AAA TAA TGT ACG GK GAG ATG CAT GA 3′) and 122 (5′ GGG TTC GAT TGG GGT TGG TGT-3′) which anneal to the four conserved regions of *T. cruzi* minicircles as described^27^. A sample of 5 μL of the elution of the extract of kissing bug intestine and feces was used as DNA template in 50 μL of final volume. Each experiment included a blank that contained water instead of DNA and a positive control that contained purified kinetoplast DNA of *T. cruzi*. The minicircle hypervariable region PCR product of 330 bp was analyzed by electrophoresis in a 2% agarose gel and visualized by GelRed staining (For more information about methodology see ESM1-Fig. S1). A sample was considered positive when at least one of the three assays showed amplification. DNA concentration was measured on all *T. cruzi* negative samples to verify extraction success (See detailed information in ESM2-Excel file).

### Statistical analyses

Generalized linear models (GLM hereafter) with identity link function were employed to assess the effect of variables on vector abundance and infection risk (measured as infected vector abundance). We constructed separated models to account for vector abundance and infection risk using the mean temperature of the warmest trimester, total annual precipitation, total vegetation cover, bromeliad cover, and the mean availability of each vertebrate group (i.e., mean records of: total native mammal species, *O. cuniculus*, total domestic mammal species, total reptile species and total bird species) as predictor variables. The model structure providing the lowest Akaike Information Criterion (AIC)^28^ was chosen in each case using the “stepAIC” function, included in the “MASS” library of the R software^29^. Models were validated in the R software: residual normality was checked using graphic methods and the Shapiro-Wilk test, and homocedasticity assumption was validated with graphic methods^30^.

### Ethical approval

All applicable institutional and/or national guidelines for the care and use of animals were followed.

## Results

**Figure 3:**
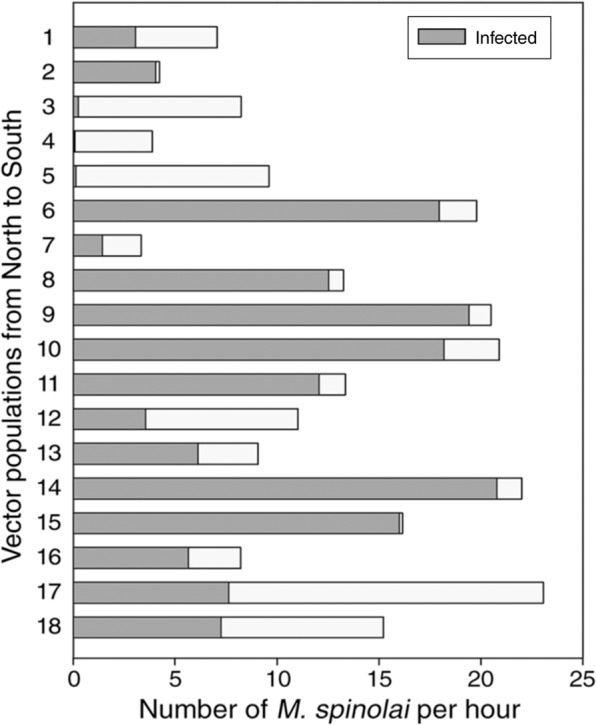
Total number of *Mepraia spinolai* (whole bar) in the 18 sampled populations. The number of infected individuals per hour are depicted in dark grey.

A total of 3044 *M. spinolai* were collected from the 18 populations. Localities were highly variables in their habitat features, vertebrate composition and availability, vector abundance and infection risk. Kissing bug abundance ranged from 3.3 to 23.1 individuals/hour, and *T. cruzi*-infection risk ranged from 0 to 20.8 infected *M. spinolai*/hour (See electrophoresis results of a subset of the samples in EMS1-Fig. S1; Fig. 3). The mean temperature of the warmest trimester (TWT) ranged from 15.9 to 20.0 °C and total annual precipitation (TAP) from 40.4 to 421.6 mm. Vegetation cover (VC) ranged from 19.1 to 78.9% and *Puya* sp. (BC) were detected in 10 out of 18 populations, covering from 1.0 to 42.9% (See detailed information per population in Table 1). Reptile availability (RA) ranged from 0 to 11.3 records/month and bird availability (BA) from 0 to 36.0 records/month. The total availability of native mammals (NMA) ranged from 0.7 to 196.7 records/month, and domestic mammals (DMA) from 0 to 6.7 records/month. The rabbit *O. cuniculus* (OCA) was present in eight populations, with a range of 0 to 8.3 records/month. See Tables 2–4 for complete lists of recorded mammal, bird and reptile species, respectively.

**Table 2:** Mammal species detected by camera traps in the 18 populations of Mepraia spinolai. *Introduced species.

Taxon	Population																	
	1	2	3	4	5	6	7	8	9	10	11	12	13	14	15	16	17	18
**Didelphimorphia**																		
*Thylamys elegans*	x					x	x	x		x	x	x	x	x	x	x	x	
**Artriodactyla**																		
Bos Taurus*																x		
Capra hircus*						x		x				x					x	
Ovis aries*						x											x	
**Carnivora**																		
Canis familiaris*			x								x			x				x
Felis silvestris*									x					x				x
*Galictis cuja*			x															
*Leopardus colocolo*											x							
*Lycalopex culpaeus*			x						x	x	x	x		x	x		x	
*Puma concolor*																x		
**Lagomorpha**																		
Oryctolagus cuniculus*			x			x					x	x	x	x	x		x	x
**Rodentia**																		
*Abrocoma benetti*											x				x	x		
*Abrothrix olivaceus*						x					x				x	x		
*Octodon degus*				x			x	x							x			
*Octodon lunatus*					x	x									x	x		
*Phyllotis darwini*	x	x	x	x	x	x		x		x	x	x	x	x	x	x	x	x

**Table 3:** Bird species detected by camera traps in the 18 populations of *Mepraia spinolai*.

Taxon	Population																	
	1	2	3	4	5	6	7	8	9	10	11	12	13	14	15	16	17	18
**Caprimulgiformes**																		
*Systellura longirostris*												x				x		
**Columbiformes**																		
*Zenaida auriculata*												x			x	x		x
**Galliformes**																		
*Callipepla californica*											x	x	x	x	x	x	x	x
**Passeriformes**																		
*Asthenes humicola*			x			x		x		x		x	x		x		x	
*Curaeus curaeus*											x							
*Diuca diuca*						x						x						
*Mimus thenca*		x						x			x	x						x
*Ochetorhynchus melanurus*					x			x	x			x			x	x		
*Phrygilus fruticeti*						x					x	x						x
*Phrygilus gayi*											x					x		
*Pteroptochos megapodius*			x		x			x		x	x	x	x	x	x	x	x	x
*Scelorchilus albicollis*			x				x				x			x	x			
*Sturnella loyca*																	x	
*Troglodytes aedon*	x	x				x					x				x			
*Turdus falcklandii*											x							
*Xolmis pyrope*											x							
*Zonotrichia capensis*						x					x	x	x					x
**Tinamiformes**																		
*Nothoprocta perdicaria*											x		x		x		x	x

**Table 4:** Reptile species detected by camera traps in the 18 populations of *Mepraia spinolai*.

Taxon	Population																	
	1	2	3	4	5	6	7	8	9	10	11	12	13	14	15	16	17	18
**Squamata**																		
*Callopistes maculatus*								x	x			x	x					
*Liolaemus fuscus*							x				x			x	x			
*Liolaemus monticola*															x	x		
*Liolaemus nitidus*			x			x		x		x					x			
*Liolaemus platei*	x	x	x					x	x									
*Liolaemus pseudolemniscatus*							x											
*Philodryas chamissonis*	x																	

The best model for kissing bug abundance, included mean temperature of the warmest trimester, total annual precipitation, vegetation cover, *O. cuniculus* availability and bird availability as predictor variables (Table 5). Significant positive estimates were detected for vegetation cover, mean temperature of the warmest trimester, and availability of the free-ranging European rabbit *O. cuniculus*. The best model for infection risk included mean temperature of the warmest trimester, bromeliad cover, total domestic mammal availability, and reptile availability as predictor variables (Table 6). Significant positive effects were detected for mean temperature of the warmest trimester, bromeliad cover and reptile availability, and negative for total domestic mammals.

**Table 5:** Generalized linear model testing the relationship between *Mepraia spinolai* abundance and the predictor variables.

Model						AIC
MS/h = Intercept + TAP + TWT + VC + BC + RA + BA + NMA + DMA + OCA						100.55
MS/h = Intercept + TAP + TWT + VC + OCA + BA						94.5
**Response**	**Variables**	**Estimates**	**SE**	***p***	**r²**	***p***
MS/h					0.82	<**0.001**
	Intercept	−40.259	12.295	**0.007**		
	TAP	−0.016	0.008	0.055		
	TWT	2.331	0.686	**0.005**		
	VC	27.409	4.144	<**0.001**		
	OCA	0.822	0.358	**0.041**		
	BA	−0.162	0.086	0.083		

MS/h: total number of *M. spinolai* per hour; TAP: total annual precipitation; TWT: mean temperature of the warmest trimester; VC: vegetation cover; BC: cover of bromeliads; RA: mean records of reptiles per month; BA: mean records of birds per month; NMA: mean records of native mammals per month; DMA: mean records of domestic mammals per month; OCA: mean records of *Oryctolagus cuniculus* per month. The complete model is shown in the top. The selected model with the lowest AIC value is shown in the bottom. Statistically significant *p*-values in bold.

**Table 6:** Generalized linear model testing the relationship between *Trypanosoma cruzi* infection risk (i.e., infected vector abundance) and the predictor variables.

Model						AIC
IMS/h = Intercept + TAP + TWT + VC + BC + RA + BA + NMA + DMA +OCA						117.34
IMS/h = Intercept + TWT + BC + NMA + DMA + RA						110.77
**Response**	**Variables**	**Estimates**	**SE**	***p***	**r²**	***p***
IMS/h					0.63	**0.003**
	Intercept	−79.133	22.023	**0.004**		
	TWT	4.581	1.199	**0.002**		
	BC	33.024	9.886	**0.006**		
	NMA	−0.072	0.040	0.096		
	DMA	−1.991	0.742	**0.020**		
	RA	1.723	0.621	**0.017**		

IMS/h: number of infected *M. spinolai* per hour; TAP: total annual precipitation; TWT: mean temperature of the warmest trimester; VC: vegetation cover; BC: cover of bromeliads; RA: mean records of reptiles per month; BA: mean records of birds per month; NMA: mean records of native mammals per month; DMA: mean records of domestic mammals per month; OCA: mean records of *Oryctolagus cuniculus* per month. The complete model is shown in the top. The selected model with the lowest AIC value is shown in the bottom. Statistically significant *p*-values in bold.

## Discussion

In this study, we examined the vertebrate hosts and habitat features associated with kissing bug abundance and infection risk at regional scale. The results showed that temperature, vegetation cover and the European rabbit availability affected positively vector abundance. On the other hand, the presence of *T. cruzi* in vector populations was associated positively to temperature, bromeliad cover and reptile availability, but negatively to the total domestic mammal availability.

Rabbit availability accounted for considerable variation in kissing bug abundance, and this association may relate to the fact this mammal is a highly accessible blood meal to *M. spinolai*. This suggestion is supported by two facts: (i) rabbit burrows frequently occur next to *M. spinolai* colonies^18^, and (ii) rabbits have mostly nocturnal habits while *M. spinolai* is a predominantly diurnal species, therefore, facilitating feeding activity on resting rabbits^20^. Furthermore, when fed separately on different mammal species under laboratory conditions, kissing bugs reach the highest fecundity when fed on rabbits^31^. Our result stresses the importance of the alien European rabbit, described as a plague in the Mediterranean-type ecosystem of South American^20^, as a critical feeding resource for kissing bug populations^32^.

Total vegetation cover influenced positively vector abundance. It is likely that, as reported in other kissing bugs^33^ and ticks (e.g., *Ixodes rictus*, the vector of Lyme borreliosis)^34^, vegetation provides shelter and stable abiotic conditions allowing high population growth rates. Some rodent species such as *Octodon degus* and *Phyllotis darwini* use thorny shrubs as permanent refuges, which increases their susceptibility to parasitism by kissing bugs. Indeed, these rodents constitute an important proportion of *M. spinolai* diet^18^, suggesting that these microsites represent critical patches for vector establishment and population growth. However, it has also been described that when *M. spinolai* is associated with rural housing, they feed on domestic animals such as dogs, cats, goats, among others^35^.

The warmest temperature showed a positive association with vector abundance, which is consistent with reports describing that insect mortality increases at low temperatures as most insects need an optimal temperature to complete their development, above which survival can decline^24,36,37^. Temperature has been described as playing an important role on kissing bug’s behavior, since it regulates essential processes in their biological cycles, such as feeding, dispersal, molting time and reproduction, all of them ultimately affecting population abundances^21,23^. We suggest that the critical thermal maximum is not approached at our sites and this might be relevant to predict changes in kissing bug abundance with a warming climate.

The total availability of domestic mammals was negatively related with infected vector abundance (i.e., infection risk). One potential explanation for this pattern is based on a simple host numerical effect assuming an opportunistic vector feeding behavior. If infected and/or more competent mammals are less abundant, reducing their contact rates with vectors, the net result will be a lower density of infected vectors. For instance, some mammal species might be less competent than others in acquiring and transmitting *T. cruzi* to vectors because of a lack common evolutionary history with the protozoan (e.g., domestic mammals). Some host species-specific features that prevent parasite inoculation or settlement are skin thickness, fur density and length, grooming behavior, repulsion behavior to kissing bug bites^38^. We suggest that Chagas disease risk should be assessed considering all the complexity associated to this multi-host system and evaluated in a wide spatial scale.

Surprisingly, reptile availability was a good predictor of *T. cruzi*-infected vector abundance (i.e., infection risk). The reptile species, mostly lizards, cohabitating with *M. spinolai* are mainly insectivorous (e.g., *Liolaemus platei*, *L. fuscus*, *L. monticola*, *L. nitidus*)^39^, which suggests they probably include kissing bugs in their diet^40^. The higher infection risk observed at increasing lizard availability can be tentatively explained if lizards become infected and amplify *T. cruzi*, which is consistent with a previous report in the North American lizard *Gerrhonotus multicarinatus webbii*^41^. There is evidence that *Mepraia* species feed on reptiles^17^ and probably lizards prey on kissing bugs^40^; therefore, lizards could become infected by vectorial transmission during thermoregulation activities, when reptiles are largely inactive, or by infected kissing bug consumption (i.e., oral transmission). Further research is needed to test the mechanistic role of lizards in the wild transmission cycle of *T. cruzi*.

Temperature and bromeliad cover were relevant habitat features positively associated with the protozoan-infection risk. Laboratory evidence indicates that *T. cruzi* replication in triatomines tends to increase with temperature up to an optimum ca. 27–30 °C^42,43^, which is in line with our regional findings. Regarding the positive contribution of bromeliad cover to *T. cruzi* infection, it is likely that this association is mediated through changes in blood meal sources such as rodent availability. Vegetation (and thus shelter) is scarce and often constitutes a limiting resource in semiarid Mediterranean-type environments. In this situation, bromeliads may provide shelter and appropriate thermal conditions to small mammals, creating kissing bug aggregations and overcrowding. As kissing bug feces contain aggregation pheromones, this may increase disproportionally disease transmission rate within colonies. Horizontal transmission through blood stealing and coprophagy has been reported in dense triatomine colonies^44,45^, and suggested in *M. spinolai*^7^, which may help to understand the mechanisms underlying the positive association between bromeliad cover and infection risk across populations.

In summary, the free-ranging European rabbit was identified as an important species accounting for variation in vector abundance across populations, suggesting this mammal species plays a key role in regulating vector abundance and, therefore, parasite transmission^37^. Infection risk, in turn, increased with lizards but decreased with the availability of domestic mammals. While the mechanisms involved in the geographical patterns here described remain to be tested, our results showing the relevance of bromeliads and temperature, and more importantly the role of lizards and the invasive European rabbit in the epidemiology of Chagas disease, contribute to a more complete understanding of the factors involved in the transmission of one of the major neglected infectious diseases^46^.

## Supplementary information

Dataset 1.

Dataset 2.
