# Supplementary material for: Lizards and rabbits may increase Chagas infection risk in the Mediterranean-type ecosystem of South America
Source: Sci Rep. 2020 Feb 5;10:1853. doi: 10.1038/s41598-020-59054-8 (PMC7002642; doi:10.1038/s41598-020-59054-8)
Supplement: Supplementary file 1 — Dataset 1. [file 41598_2020_59054_MOESM1_ESM.docx]

Lizards and rabbits may increase Chagas infection risk in the Mediterranean-type ecosystem of South America.

Esteban San Juan, Raúl Araya-Donoso, Alejandra Sandoval-Rodríguez, Andrea Yáñez-Meza, Nicol Quiroga and Carezza Botto-Mahan.

Figure S1. Agarose gels for 121 and 122 PCR products of *Trypanosoma cruzi* from a subset of samples of each *Mepraia spinolai* population (8 samples per population). LAD: ladder, NTC: negative template control, POS: positive amplification control (i.e., *T*. *cruzi* DNA). Each number corresponds to one of the 18 sampled *M. spinolai* populations. *Trypanosoma cruzi* minicircles (approx. 1400 bp) are composed of four conserved sequence regions (the replication origin, each one of approx. 120 bp), and four variable sequence regions (each one of approx. 250bp), which are alternated. The PCR assay developed to amplify *T. cruzi* minicircles use two oligonucleotides, which anneal with the conserved sequence regions in a way that an amplicon including small parts of the conserved region (40 bp approx.) plus the size of one variable region is obtained (approx. 330bp). This assay when the amount of minicircle DNA is high, generates an additional band of higher size (approx. 700 bp). This amplicon includes two variable regions, one constant region plus small segments of the constant regions where the oligonucleotides align (a dimer). The results show that the positive control with high DNA as template generate these two bands. Positive *M. spinolai* samples give the small amplicon but some give the double bands described before (Wincker et al. 1994, Breniere et al. 1992). Gel photographs were not cropped and over-exposed in the editing process. Information on gel photographs (e.g., numbers, abbreviations, and horizontal brackets) were done with Adobe Illustrator CC2019. Photograph by Alejandra Sandoval-Rodríguez.


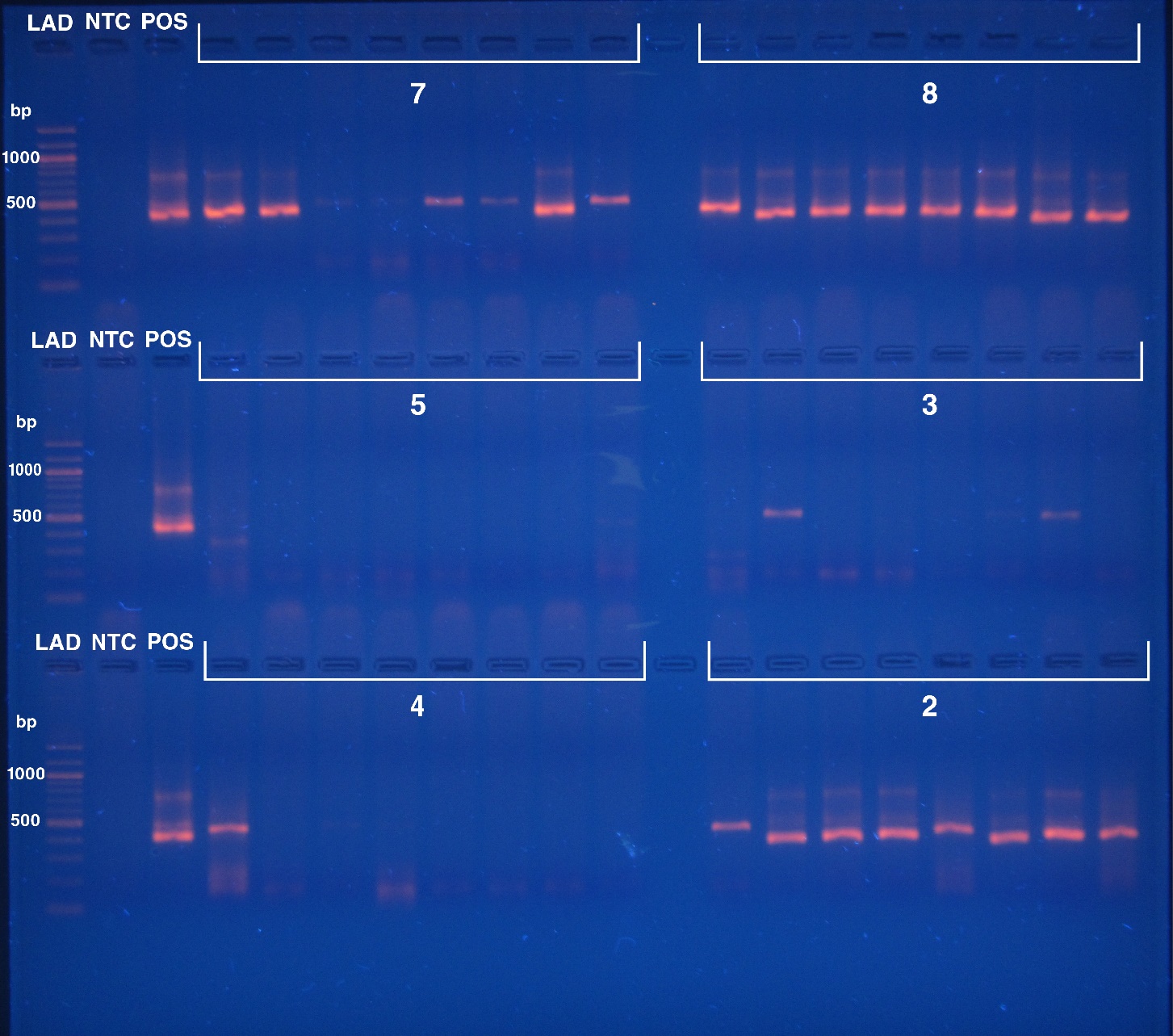


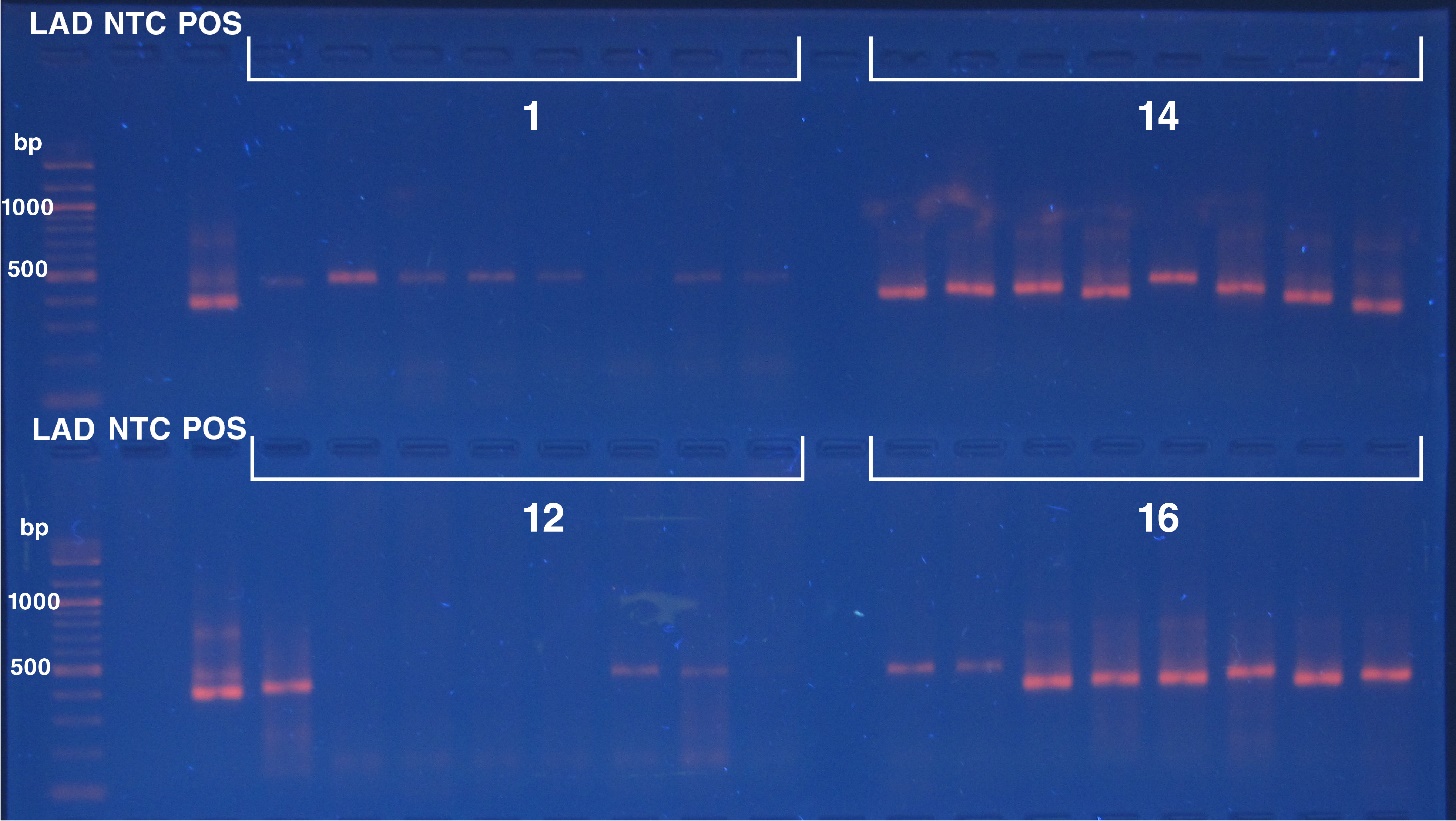


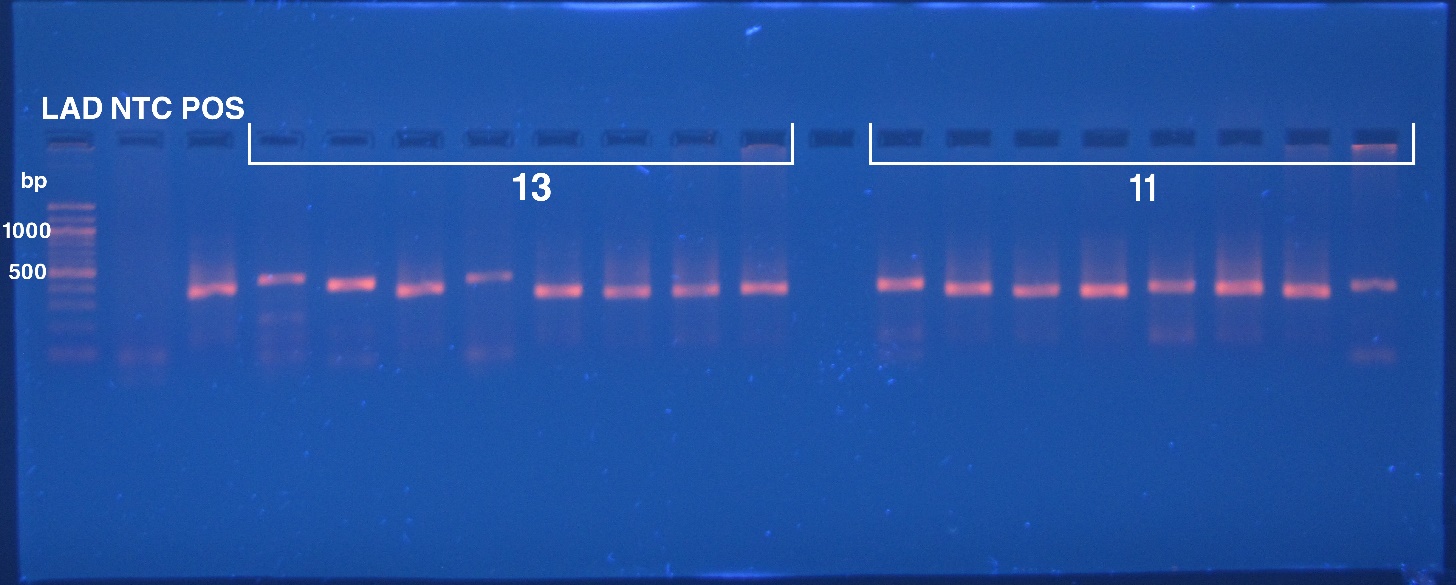


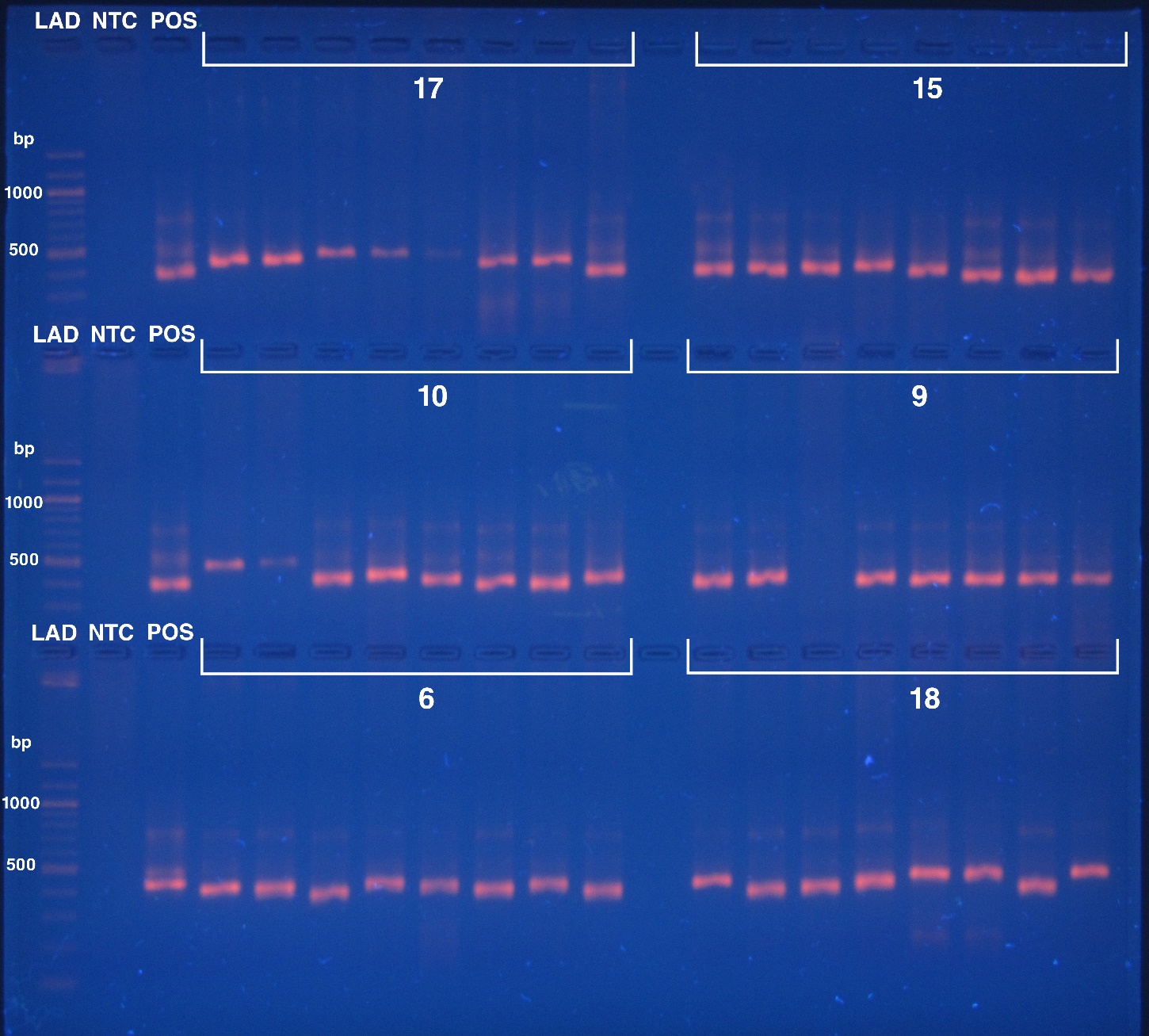


References

Breniere SF, Bosseno MF, Revollo S, Rivera MT, Carlier Y & Tibayrenc M. 1992. Direct identification of *Trypanosoma cruzi* natural clones in vectors and mammalian hosts by polymerase chain reaction amplification. American Journal of Tropical Medicine Hygiene 46: 335-341.

Wincker P, Bosseno M-F, Britto C, Yaksic N, Cardoso MA, Morel CM & Brenière SF. 1994. High correlation between Chagas’ disease serology and PCR-based detection of *Trypanosoma cruzi* kinetoplast DNA in Bolivian children living in an endemic area. FEMS Microbiology Letters 124: 419-424.
